# Chemical and transcriptional responses of Norway spruce genotypes with different susceptibility to *Heterobasidion *spp. infection

**DOI:** 10.1186/1471-2229-11-154

**Published:** 2011-11-08

**Authors:** Marie Danielsson, Karl Lundén, Malin Elfstrand, Jiang Hu, Tao Zhao, Jenny Arnerup, Katarina Ihrmark, Gunilla Swedjemark, Anna-Karin Borg-Karlson, Jan Stenlid

**Affiliations:** 1 Ecological Chemistry Group, Department of Chemistry, KTH, Sweden; 2 Department of Forest Mycology and Plant Pathology, Swedish University of Agricultural Sciences, Sweden

## Abstract

**Background:**

Norway spruce [*Picea abies *(L.) Karst.] is one of the most important conifer species in Europe. The wood is economically important and infections by wood-rotting fungi cause substantial losses to the industry.

The first line of defence in a Norway spruce tree is the bark. It is a very efficient barrier against infection based on its mechanical and chemical properties. Once an injury or an infection is recognized by the tree, induced defences are activated. In this study we examined transcriptional response, using 454-sequencing, and chemical profiles in bark of Norway spruce trees with different susceptibility to *Heterobasidion annosum s.l. *infection. The aim was to find associations between the transcriptome and chemical profiles to the level of susceptibility to *Heterobasidion *spp. in Norway spruce genotypes.

**Results:**

Both terpene and phenol compositions were analysed and at 28 days post inoculation (dpi) high levels of 3-carene was produced in response to *H. annosum*. However, significant patterns relating to inoculation or to genotypes with higher or lower susceptibility could only be found in the phenol fraction. The levels of the flavonoid catechin, which is polymerized into proanthocyanidins (PA), showed a temporal variation; it accumulated between 5 and 15 dpi in response to *H. annosum *infection in the less susceptible genotypes. The transcriptome data suggested that the accumulation of free catechin was preceded by an induction of genes in the flavonoid and PA biosynthesis pathway such as *leucoanthocyanidin reductase*. Quantitative PCR analyses verified the induction of genes in the phenylpropanoid and flavonoid pathway. The qPCR data also highlighted genotype-dependent differences in the transcriptional regulation of these pathways.

**Conclusions:**

The varying dynamics in transcriptional and chemical patterns displayed by the less susceptible genotypes suggest that there is a genotypic variation in successful spruce defence strategies against *Heterobasidion*. However, both high levels of piceasides and flavonoids in the less susceptible genotypes suggested the importance of the phenolic compounds in the defence. Clearly an extended comparison of the transcriptional responses in the interaction with *Heterobasidion *between several independent genotypes exhibiting reduced susceptibility is needed to catalogue mechanisms of successful host defence strategies.

## Background

Norway spruce [*Picea abies *(L.) Karst.] is one of the most important conifer species in forest ecosystems both ecologically and economically in Europe. Being long-lived organisms, spruce trees rely on both induced and constitutive defences to restrict the spread of invading fungi and insects. The first line of defence in a Norway spruce trees is the bark. The combination of the physical properties of tough lignified and suberized walls that provide a hydrophobic obstacle and the chemical properties of phenolics and terpenes makes bark a very efficient barrier against infection [1]. Once an injury or an infection is recognized by the tree, induced defences are activated, including cell wall re-enforcements, production of lytic enzymes and secondary metabolites such as phenols, stilbenes, lignans, flavonoids, and terpenes [1-4].

The root-rot fungus *Heterobasidion *spp. species complex is the most serious pathogen on Norway spruce in Scandinavia [5] causing root and stem rot and rendering the timber defective for sawing and pulping. Several studies indicate that genetically determined host characteristics partly determine the susceptibility of Norway spruce to *Heterobasidion *infections [6-11].

To protect themselves against pathogens and pests, conifers such as spruce, have evolved complex constitutive and inducible defence mechanisms [1,2]. Many of these are associated with the production of secondary metabolites to delay or stop the establishment of fungi or insects within the tree [2,12-14]. Oleoresins produced in the resin ducts in the phloem are part of the constitutive defence in the bark [15,16]. Upon attack, *de novo *differentiation of xylem resin ducts [1,15,17] and production of defence-associated terpenes are reported [15,18-20]. Similarly, swelling and proliferation of polyphenolic parenchyma cells (PP cells) in the bark [21,22] and changes in phenolic concentration [23-26] are seen in response to pathogen attack.

**Figure 1:**
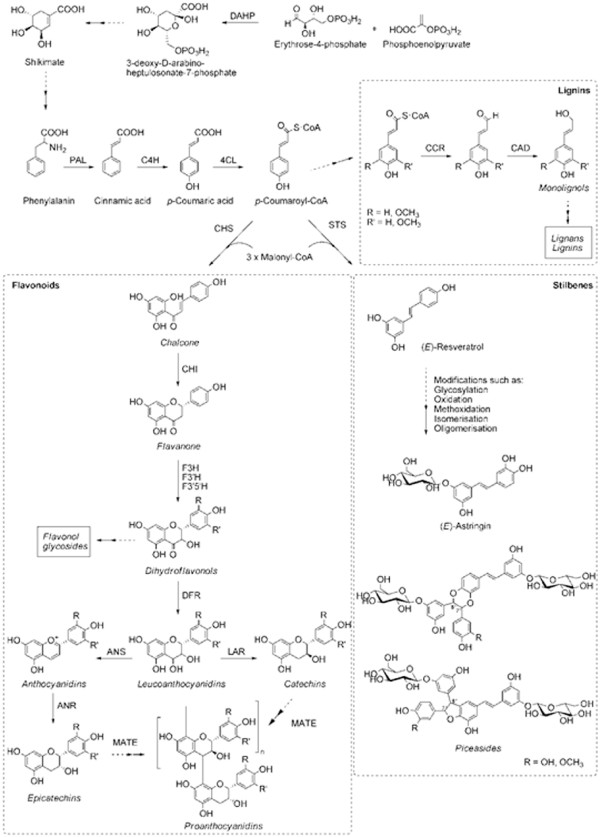
**Secondary metabolism leading to the biosynthesis of proanthocyanidines and to the biosynthesis of stilbenes**. Abbreviations: DAHP, 3-deoxy-D-arabino-heptulosonate 7-phosphate synthase; PAL, L-phenylalanine ammonia-lyase; C4H, cinnamate 4-hydroxylase; 4CL, 4-coumarate CoA-ligase; CCR, cinnamoyl-CoA reductase; CAD, cinnamyl-alcohol dehydrogenase; STS, stilbene synthase; CHS, chalcone synthase; CHI, chalcone isomerase; F3H, flavanone 3-hydroxylase; F3'H, flavanoid 3'-hydroxylase; F3'5'H, flavanoid 3'5'-hydroxylase; DFR, dihydroflavanol reductase; ANS, anthocyanidin synthase; ANR, anthocyanidin reductase; LAR, leucoanthocyanidin reductase; MATE, multidrug and toxic compound extrusion transporter. Adapted from Mellway *et al. *[30].

The regulation and biosynthesis of terpenes in the response to insect attack have been successfully explored using combinations of transcript profiling and chemical characterizations over the last decade [19,27,28]. Similar approaches have been applied on studies of flavonoids in response to leaf pathogens in poplar [29,30]. However, in spruce this type of approach has not yet been applied on the regulation and biosynthesis of phenolics in interaction with pathogens. From a metabolic point of view, plant phenolics constitute a much more heterogeneous group than terpenes. The phenolics are biosynthesized by several different routes but they all derive from products of the shikimic acid and phenylpropanoid pathways (Figure 1) [31].

Fungal infection commonly results in a decrease of phenolic glycosides and a subsequent increase of the corresponding aglycones [12,14,24,26,32]. The accumulation of aglycones could be a result of β-glucosidase activity from either the fungus [14] or the tree [33]. Possible relations between stilbene content and resistance to *Heterobasidion *spp. have been investigated and Lindberg *et al.*, [12] found that the initial concentration of the stilbene astringin was negatively correlated with the depth of the hyphal penetration in Norway spruce bark. In contrast, no correlation between constitutive bark stilbene glycosides and resistance to *H. annosum *was found in Sitka spruce (*Picea sitchensis *[(Bong.) Carrière]) [34]. Better resistance to *Ceratocystis polonica *[(Siemaszko) C. Moreau] infection has been associated with low constitutive levels of the stilbene isorhapontigenin, phenol diversity and accumulation of the flavonoid (+)-catechin in the phloem of Norway spruce after inoculation [23,25].

In this study we examined transcriptional response and chemical profiles in clonal Norway spruce trees. The clones were quantitatively scored for susceptibility to *Heterobasidion *spp. based on screening for visible decay in the stand in 2004 [7]. The present investigation was carried out in a replicate plantation in mid Sweden. For sampling we selected four genotypes (clones), two genotypes where the majority of the ramets were heavily attacked by *Heterobasidion *spp. and two genotypes that showed almost no infection, based on the analysis in the investigation in 2004.

Our aim was to find associations between the transcriptome and chemical profiles to the level of susceptibility to *Heterobasidion *spp. in Norway spruce genotypes. We found associations between the level of susceptibility and the phenol content and genotypic differences in the terpene content.

## Methods

### Plant material and sampling

The plant material was from a site that was part of a Swedish regional clonal forestry program at SkogForsk [35]. The stand was situated at Årdala, Sweden, (59°01' N, 16°49' E) and was established in 1984 with 311 genotypes as 3-year old bare root cuttings. It was planted in a Roman square design with nine replicates and single tree plots with 1.4 m spacing within main plots. The genotypes were distributed in eight clone mixtures planted in different main-plots. The selected Norway spruce genotypes have previously been classified for natural susceptibility to infections of *Heterobasidion *spp. [7].

Three ramets per clone were used and at day 0, two roots of each tree were chosen, one for inoculation and one for wounding treatment. The roots assigned to inoculation were artificially inoculated with *Heterobasidion annosum *[(Fr.) Bref.] (Sä 16-4) [36]. To allow the fungus to enter the root, three 5 mm circular wounds were made on a line perpendicular to the root elongation. Each bark disc was cut in half (parallel to root elongation). One half was put in a 2 mL microcentrifuge tube containing 1.5 mL of RNAlater (Ambion) for subsequent transcriptome profiling and the other half was placed in a vial containing 1 mL of hexane with 57 ngμL^-1 ^pentadecane as internal standard and 102 ng μL^-1 ^of the antioxidant 3-tert-butyl-4-hydorxyanisole for extraction of terpene content. Wooden plugs 5 mm in diameter and inoculated with *H. annosum*, were prepared according to Stenlid & Swedjemark [37], and attached to the wounds with Parafilm^®^. The roots assigned to wounding were handled identically except that a sterile wooden plug was attached to each wound.

After five days the left inoculation point on each root was sampled. The wooden plug was removed, and thereafter a 1.5 cm diameter bark sample was taken around the inoculation point and the bark sample was cut in half (parallel to root elongation). One half was put in a 2 mL microcentrifuge tube containing 1.5 mL of RNAlater (Ambion) for subsequent transcriptome profiling and the other half was placed in a vial containing 1 mL of hexane with 57 ng μL^-1 ^pentadecane as internal standard and 102 ng μL^-1 ^of the antioxidant 3-tert-butyl-4-hydorxyanisole for extraction of terpene content. At 15 and 28 days post inoculation (dpi) the procedure was repeated for the other two inoculation holes. At 15 dpi the inoculation point furthest to the right was collected and 28 dpi the central point was sampled. The lesion length on the wound/inoculation point harvested at 28 dpi was measured at 44 dpi, to validate that inoculation was successful as lesion lengths has been shown to correlate with fungal growth in field experiments [6,8,38].

Temperature data were collected during the sampling period (13 August - 9 September 2008) by the data logger Tinytag™ and air temperatures ranged between 6.2°C and 25.8°C.

### Chemical analyses

#### Chemicals

Acetonitrile, water and formic acid, all of LC-MS grade, were purchased from Sigma Aldrich. Hexane, methanol and water of LC grade used for extractions were bought from SDS (Val de Reuil, France). n-Pentadecane was bought from Lancaster (98% GC-purity) and 3-tert-butyl-4-hydroxyanisole (BHA, ≥ 90% GC-purity) from Fluka. Vanillyl alcohol and some of the phenol reference chemicals were synthesized in the lab at KTH; other phenol reference chemicals were received as gift from Annie Yart (INRA, Orléans, France). Terpene reference chemicals were obtained from commercial sources.

#### Preparation of samples for GC-MS and HPLC-MS analysis

The extraction of terpenes with hexane was initiated during sampling in the field and thereafter carried out in room temperature overnight. The hexane was collected for GC-MS analysis and the residue was washed again with 1 mL of hexane for 1 h. To extract phenols the hexane was removed and 0.5 mL of 80% methanol (with 106 ng μL^-1 ^of vanillyl alcohol and 108 ng μL^-1 ^BHA) was added to the sample. The extraction of phenols continued at room temperature overnight. All samples were centrifuged at 6000 rpm for 10 minutes and stored in the freezer until analysed. The residues were placed in open vials in a ventilated cupboard and further dried in 80°C for 40 hours before the samples were weighed.

#### GC-MS analyses

Hexane samples were separated on a Varian 3400 GC with a DB-wax column (30 m, 0.25 mm id and 0.15 μm film thickness, J&W Scientific, Agilent, Santa Clara, CA, USA) using the following temperature program: 40°C for 3 min, ramp with 4°C/min up to 230°C and kept constant for 19 min. Injector temperature was 225°C and the transfer line 235°C. Helium (0.69 bar inlet pressure) was used as carrier gas. The GC was connected to a Finnigan SSQ 7000 MS instrument with electron ionization (source: 150°C, 70 eV). Separations of enantiomers were performed as described by Borg-Karlson *et al. *[39].

#### HPLC-ESI-MS analyses

LC-MS analyses were performed on a Finnigan HPLC system, consisting of a Surveyor MS Pump Plus, Surveyor Autosampler Plus and Surveyor PDA Plus detector, coupled to a 2D linear ion trap, Finnigan LXQ (Thermo Fisher Scientific, San José, CA, USA).

An Ascentis express RP-amide column (15 cm, 2.1 mm i.d., 2.7 μm film thickness; Supelco, Bellefonte, PA, USA) together with an RP-amide guard column (2 cm, 2.1 mm i.d., 5 μm film thickness, Supelco, Bellefonte, PA, USA) was used for HPLC separations. The separation was carried out with a gradient of 0.1% formic acid in water (A) and 0.1% of formic acid in acetonitrile (B) and flow rate 200 μL/min, oven temperature was 30°C. The elution gradient was as follows (% of B): 10% (0-3 min), 10-30% (3-51 min), 30-100% (51-57 min), hold for 11 min and finally decrease to 10% B during 2 min. The system was allowed to equilibrate for 20 min between analyses.

All measurements were performed in negative mode with full scans ranging between m/z 50-1000. The ESI source was optimized on isorhapontigenin and set up as follows: source voltage 4.00 kV, capillary temperature 270°C, sheet gas flow 40 au (arbitrary unit) and sweep gas 20 au. The capillary voltage was set to -23.00 V and the tube lens to -109.80 V.

### Transcript profiling

#### RNA extraction, cDNA synthesis and sequencing

Total RNA was isolated essentially as described by Chang *et al. *[40]. To eliminate contamination of genomic DNA the total RNA was treated with DNaseI (SIGMA) before use. RNA quality and quantity was assessed with an RNA Nano assay on a Bioanalyzer 2100 (Agilent). Poly(A)+RNA was extracted from the samples with the Dynabeads^® ^mRNA Purification Kit (Invitrogen) according to the manufacturer's instructions. The purified mRNA was amplified with the MessageAmpIII kit (Ambion) according to the manufacturer's instruction. First strand cDNA was synthesized from the amplified RNA (aRNA) using the iScript cDNA Synthesis Kit (Bio-Rad) according to the protocol supplied by the manufacturer except that the RT-reaction was allowed to proceed over 50 minutes. Second strand synthesis was performed as described by Sambrook and Russel [41] using enzymes purchased from Fermentas. Double stranded cDNA of sufficient quality was pooled according to genotype and treatment.

Two to five μg each of 24 cDNA samples representing all time points and treatments were submitted for template preparation and pyrosequencing on a GS FLX (Roche, 454) at the Norwegian Sequencing Centre http://www.sequencing.uio.no, according to themanufacturer protocols (Roche Applied Science). Sequence reads and quality scores for sequences were obtained from the Norwegian Sequencing Centre.

#### Verification of gene expression by qPCR

Purified aRNA (1 μg) from all four genotypes (2405, 7398, 3178 and 3340) were reverse transcribed with the iScript™ cDNA synthesis kit (Bio-Rad). The cDNA synthesis was diluted 1:1 in deionizer water, and an aliquot of cDNA equivalent of 25 ng of aRNA was used per 20 μL of PCR reaction using Maxima^® ^SYBR Green/Fluorescein qPCR Master Mix kit (Fermentas) and a final concentration of 0.5 μM of each primer. Primers were designed from isotig sequences using the Primer3 software [42] with a melting temperature (Tm) between 58°C and 60°C, and amplicon length between 95-183 bp (Additional file 1). The thermal-cycling condition parameters, run on a iQ™5 Multicolor Real-Time PCR Detection System (Bio-Rad), were as follows: 95°C for 10 min; 40 cycles of 95°C for 15 sec, 58 or 60°C for 10 sec and 60°C for 1 min. Each run was followed by a melt curve analysis to validate the specificity of the reaction. A linear plasmid standard curve was used to measure the PCR efficiency and primer pairs with efficiency lower than 95% was discarded. Two technical replicates were prepared for each sample.

Transcript abundance was normalized to the reference genes *phosphoglucomutase *[43], *eukaryotic translation initiation factor 4A *(*elF4A*) [44]*and elongation factor 1-α *(*ELF1α*). The relative expression was calculated using REST 2006 [45].

### Bioinformatics and statistical analyses

The sequences retrieved were assembled with the sequence assembler software Newbler v2.3 (Roche) http://my454.com/ with default settings for cDNA assembly with the sff-files as input file. The sequence assembly was carried out on the freely available Bioportal http://www.bioportal.uio.no. The combined sequences from all treatments were assembled into the gene-equivalent isogroups and the plausible splice variants, isotigs. For a detailed explanation of the terms isogroup, isotig and their connection with contigs see Ewen-Campen [46] but generally an isogroup should equal a gene, isotigs should correspond to splice variants thereof and contigs to exons. Contigs were subjected to visual inspection in ace format with the software Tablet [47]. The combined assembled sequences from all libraries was used as a reference file and were annotated with the software Blast2GO [48], where the sequences got annotated to BLASTx homologies, GO terms and EC numbers as well as scanned with InterProScan. Furthermore, the data set was trimmed for fungal sequences by identification of species belonging based on the BLAST homologies with MEGAN [49].

In order to get an estimate of relative gene expression between the libraries, count data of the occurrence of the expressed genes in the individual samples were retrieved by assembling individual reads from each library with the isogroups and isotigs in the reference file as a reference. The count data were aligned in R and imported into the R-package DESeq [50] and normalized on number of counts and subjected to further pair-wise differential expression transcriptome analysis.

The normalized count data were transformed to homoscedastic data in DESeq and clustered with JMP™ by Ward's hierarchical cluster. The contigs annotated into pathways leading to production of terpenes, stilbenes and proanthocyanidins were clustered separately.

R (The R Foundation for Statistical Computing, TU Wien, Vienna, Austria) was used for ANOVA of lesion length. Multivariate analyses were performed with the software CANOCO (Version 4.54, developed by Cajo J. F. Ter Braak and Petr Smilauer, Biometris Plant Research International, The Netherlands). Variables were subjected to log transformation, unit variance scaling and mean centring prior to ordination. Differences in constitutive concentrations of terpenes and phenols were evaluated by t-test assuming unequal variance. Comparisons of concentrations before and after treatments were made by pairwise t-tests on samples from the same root. The t-tests were carried out with the data analysis tool in Excel (Microsoft) after log transformation.

## Results

### Inoculation

Lesion lengths at 44 dpi were significantly longer after inoculation than after wounding alone (ANOVA, two factor with replication: *p = *0.01) (Table 1). However, no significant differences in lesions lengths could be found between genotypes (*p = *0.36).

**Table 1:** Average lesion lengths (mm) (+/- SD) for wounded and inoculated roots.

Genotype	Susceptibility*	Wounded	Inoculated
2405	LS	25 (0.6)	33 (11)
7398	LS	23 (1.7)	42 (25)
3178	HS	24 (1.7)	39 (8)
3340	HS	23 (2.6)	67 (47)

Measured 44 dpi at the point sampled at 28 dpi, n = 3.

*Highly (HS) or less (LS) susceptible according to Swedjemark [8]

### Assembly

The four sequenced genotypes rendered 492,102 reads in total and these were unevenly distributed between the samples (Additional file 2). The sequences were assembled and the resulting isotigs were automatically annotated (Table 2). As no reference genome is available for conifers we cannot estimate the percentage of the total genes that are covered in this data set but the numbers of possible unique transcripts are similar to previous conifer studies [51]. In this study we focused on isotigs associated with terpene- and phenylpropanoid biosynthesis.

**Table 2:** Transcriptome assembly and annotation statistics

Assembly		Annotation of isotigs	
Total reads	492 102	Nr. with BLASTx	13 390
Total bases	146391859	homology	
Assembled reads	242 206	Nr. GO Annotated	8 468
Inferred read error	1.51%	GO Annotations	41 330
Q40*	94.53%	Nr. with KEGG EC	3 605
Singletons	5 5334	Total KEGG EC Nr.	45 183
Isogroups ("genes")	9 678	Nr. InterproscanTotal	79 194
Isotigs ("transcripts")	14 364		
Isotig N50	769		
Mean no. isotigs per isogroup	1.5		
Isogroups with one Isotig	7239		
Contigs ("exons")	17228		
Mean no. contigs per isotig	2		

* Q40 of contigs of at least 500 bp length

### Phenols and phenylpropanoid biosynthetic pathway

**Figure 2:**
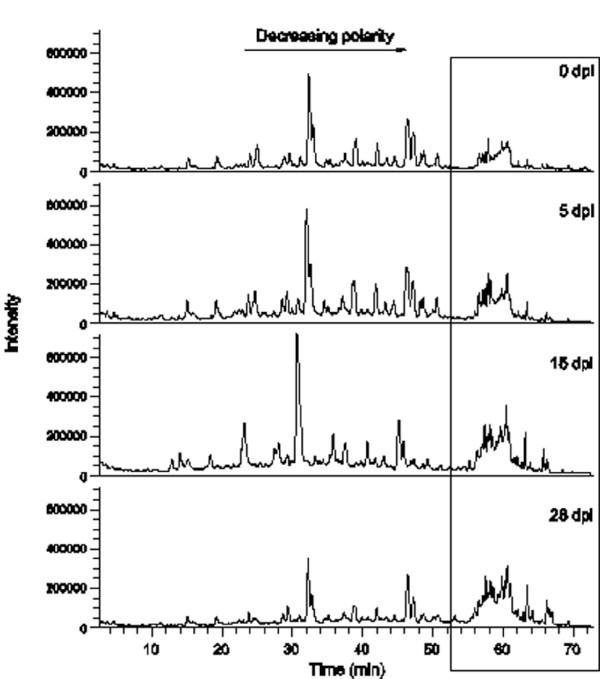
**LC-MS chromatograms for samples taken from the same *H. annosum*-inoculated root 0, 5, 15 and 28 days post inoculation**. The box enhances the area where compounds with relatively low polarity elute.

**Figure 3:**
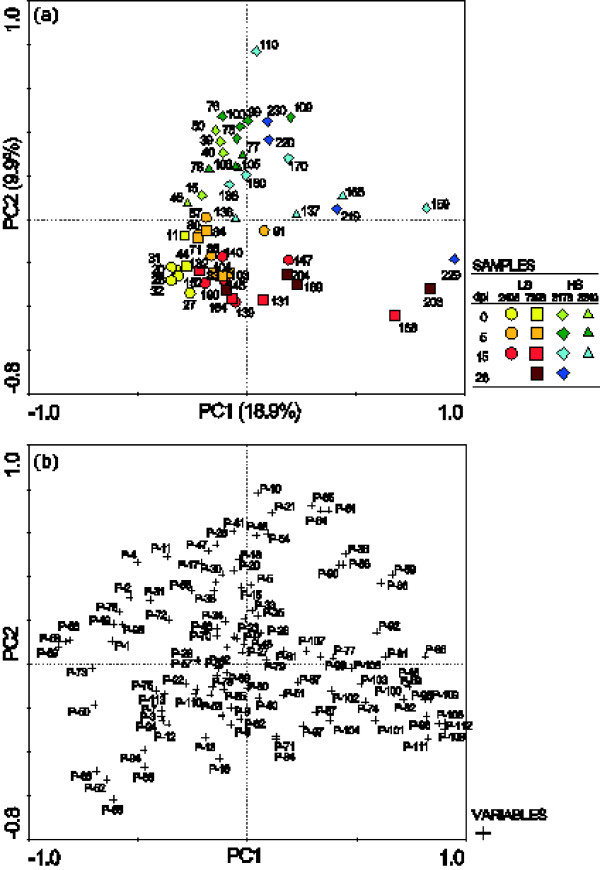
**PCA based on relative phenol composition**. (a) Sample score plot. Odd numbers: inoculated samples, even numbers: wounded samples. Less susceptible clones (LS; 2405, circles and 7398, squares) are coloured in yellow-red and highly susceptible clones (HS; 3178, diamonds and 3340, triangles) are coloured in green-blue. The percentages of the axes states how much of the variation the PC explain. (b) Corresponding variable loading plot. The constitutive levels of P-24 (unknown glucoside), P-52 (piceaside A/B) and P-66 (piceaside G/H) were higher in samples from less susceptible clones. Further information on phenolic numbering (P-#) is found in Additional file 3.

**Figure 4:**
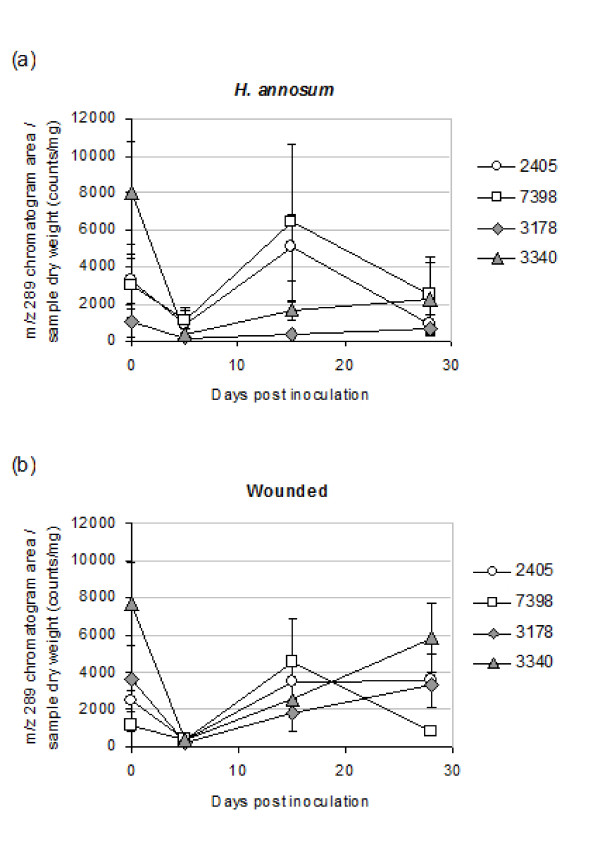
**Average levels of (+)-catechin in *H. annosum *inoculated (a) and wounded bark (b) at 0, 5, 15 and 28 dpi**. Error bar indicates SE.

Among the constitutive phenols, two astringin dimers (piceaside A/B and G/H) and one unknown phenol glucoside were found in higher concentration in bark from less susceptible genotypes (*p *< 0.05). The most obvious effect on phenol content caused by inoculation was the decrease of polar substances eluting early in the chromatogram and an increase of the late eluting, less polar compounds (Figure 2). Figure 3 shows a PCA based on the relative phenol composition of the samples. The first PC explained 19% of the variation and mainly separated the samples on time and treatment. Samples taken from the roots on the day of inoculation were placed to the left in the plot and furthest to the right were samples taken from inoculated roots at 15 or 28 dpi. The second PC had a tendency to separate the high and low susceptible genotypes. The tendency was more prominent for constitutive samples and at early stages of the inoculation; samples taken from inoculated roots at 15 and 28 dpi were not separated on a susceptibility basis (Figure 3). The levels of the flavonoid catechin in the bark samples were strikingly reduced at 5 dpi in comparison to the constitutive levels. Catechin accumulated significantly between 5 and 15 dpi in both *H. annosum *inoculated (*p *= 0.024) and wounded bark (*p *= 0.003) and at 15 dpi the levels of extractable catechin were comparable to the control (Figure 4). The accumulation of free catechin was more immediate in the less susceptible genotypes in response to *H. annosum *compared to in the highly susceptible genotypes (Figure 4a*p *< 0.05, unpaired t-test). It should be noted that the pattern of extractable catechin varies among the genotypes; for example 3178 did not show any pronounced reduction of extractable catechin at 5 dpi compared to the control, while the reduction in 3340 was much more pronounced (*p *= 0.0086, unpaired t-test).

**Figure 5:**
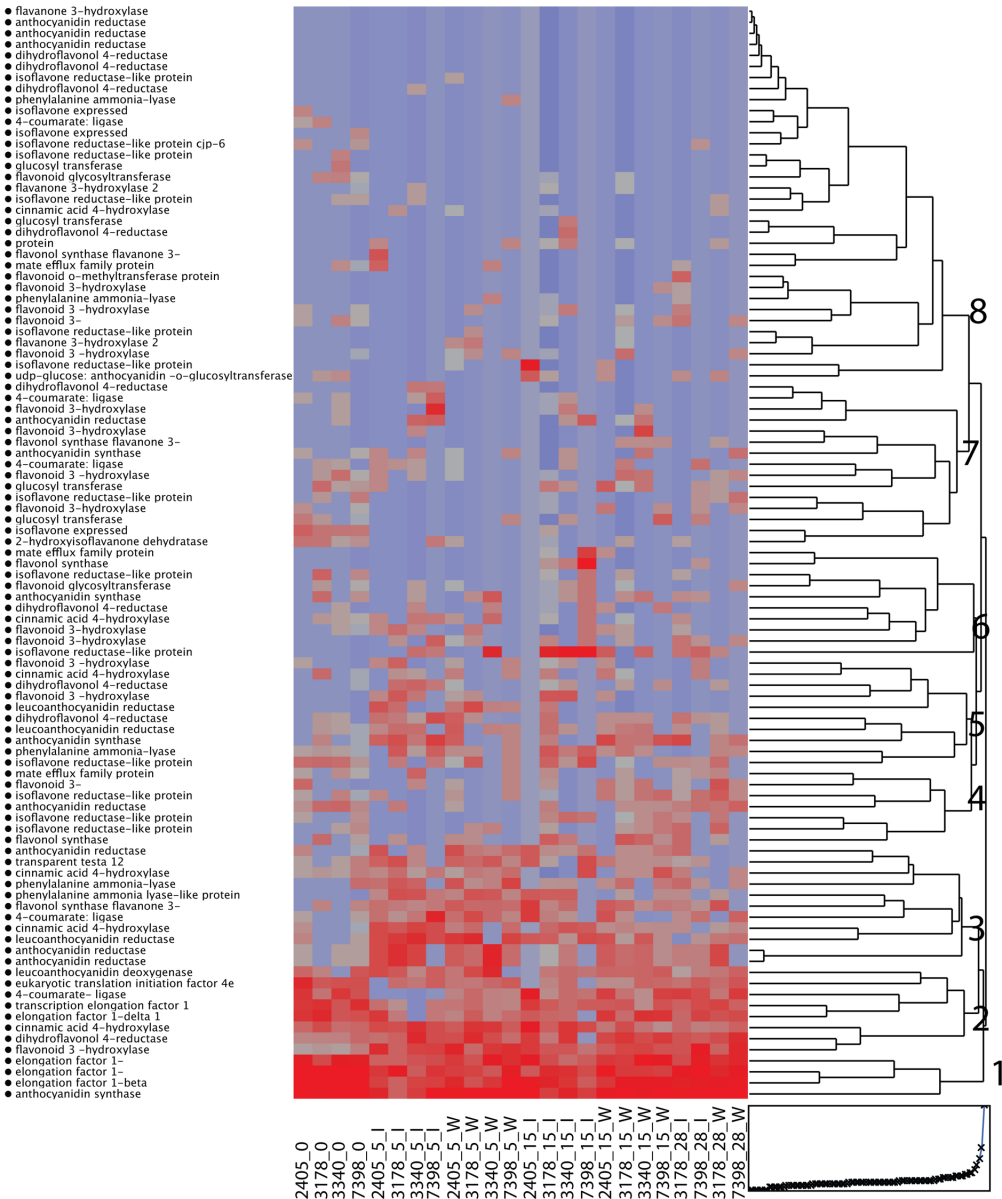
**Hierarchical clustering of the gene expression of a subset of the sequences assembled as isotigs with a BLASTx homology to genes in the flavonoid or proanthocyanidin biosynthetic pathway**. The heatmap goes from blue to red with increasing gene expression. Eight clusters (1-8) were identified as indicated in the figure. Clone number and treatment are indicated in the figure.

A cluster analysis based on the count data of isotigs (Accession number) with similarity to selected genes (*phenylalanine ammonia lyase *(*PAL*), *cinnamic acid 4-hydroxylase *(*C4H*), *4-coumarate ligase *(*4CL*), *flavanone-3-hydroxylase *(*F3H*), *dihydroflavonol-4-reductase *(*DFR*), *anthocyanidin reductase *(*ANR*), *leucoanthocyanidin reductase *(*LAR*), *MATE-like, cinnamoyl CoA reductase *(*CCR*) and *cinnamyl alcohol dehydrogenase *(*CAD*)) in the phenylpropanoid and flavonoid pathway together with selected reference genes resulted in eight clusters (cluster 1-8, Figure 5).

Cluster 1 includes the selected reference genes that are highly expressed throughout the experiment. The isotigs in cluster 2 are all very highly expressed through all treatments. Cluster 3 contain isotigs that are significantly up-regulated at 5 dpi compared to the control, irrespective of treatment, and then remain induced throughout the experiment, such as two *ANR *sequences and one *LAR *sequence (Figure 5). All of the isotigs annotated as *LAR *are significantly up-regulated at 5 dpi (*p *< 0.05) compared to the control. Cluster 4 includes isotigs which show a higher expression in the control and in wounded samples at 15 and 28 dpi. In cluster 5 and 6 isotigs activated in response to infection at 5 an 15 dpi are found, for instance isotigs annotated as *PAL*, *LAR *and *F3H *(Figure 5). Interestingly, isotigs representing the genes directly involved in lignification, i.e. isotig 14332 (*CCR*), show a transient 3-4 fold up-regulation at 5 dpi compared to the control, but a corresponding up-regulation cannot be detected at 15 or 28 dpi. None of the isotigs annotated as *CAD *show any significant regulation. Clusters 7-8 included no or very few isotigs with significant differential expression between time points.

**Figure 6:**
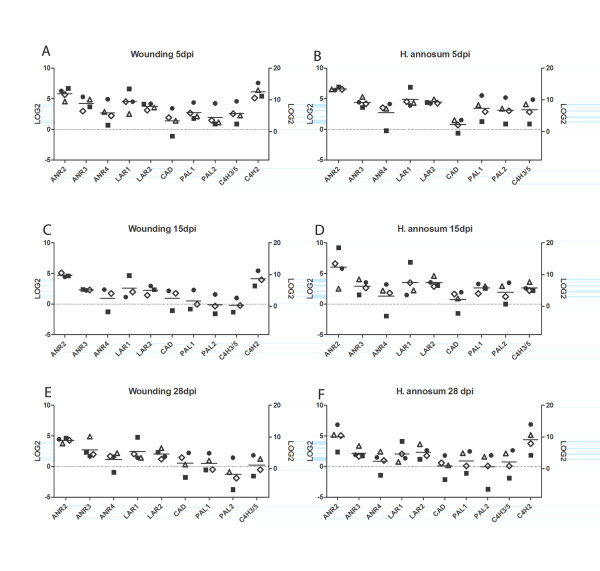
**Relative expression of selected isotigs measured by qPCR 5 dpi (a), 15 dpi (c) and 28 dpi (e) wounding treatment and 5 dpi (b), 15 dpi (d) and 28 dpi (f) *H. annosum *inoculation compared to untreated bark**. The relative expression (Log2 values) of isotigs with significant similarity to *ANR2, ANR3, ANR4, LAR1, LAR2, TT2, CAD, PAL1, PAL2 *and *C4H3/5 *are represented on the left Y-axis and the relative expression of C4H2 on the right Y-axis. Horizontal lines correspond to the average level of expression among the four genotypes. Symbols: less susceptible clones 2405 (circles) and 7398 (squares), highly susceptible clones 3178 (diamonds) and 3340 (triangles).

The overall expression pattern of the isotigs associated with the phenylpropanoid and flavonoid pathways was similar in the controls (Figure 5). At 5 dpi the general expression pattern was similar for all treatments except the isotigs found in clusters 4-6. No clear separation between highly and less susceptible genotypes could be detected at 5 or 15 dpi but the highly susceptible genotypes show more similar expression patterns after inoculation at these time points than the less susceptible genotypes (Figure 5). The observation that the less susceptible genotypes sometimes show contrasting expression patterns in response to wounding and inoculation is clearly verified in the qPCR analysis of the four genotypes (Figure 6).

The qPCR analysis of *PAL, C4H, CAD, LAR *and *ANR *genes confirmed the above picture. *PAL1, C4H*2 and *C4H3/5 *were significantly up-regulated at 5 dpi, irrespective of treatment (*p *< 0.05, Figure 6). At later time points only *C4H2 *was significantly (*p *< 0.05) up-regulated. No significant up-regulation was observed for *CAD*. Significant regulation (*p *< 0.05) was seen for *ANR2, ANR3, LAR1 *and *LAR2 *at 5 dpi (Figure 6) and all but *LAR1 *stayed up-regulated throughout the experiment. An isotig with similarity to the R2R3 myb transcription factor gene *TT2 *(*transparent testa 2*) showed a significant up-regulation for both treatments at all time points (*p *< 0.05). The actual levels of expression of the tested genes varied between genotypes: 7398 showed down regulation of *PAL1*, *PAL2, C4H3/5 *and *CAD *at 15 and 28 dpi in wounding and at 28 days post *H. annosum *inoculation while 2405, for example, did not (Figure 6c, e-f).

### Terpenes and terpenoid biosynthesis

**Figure 7:**
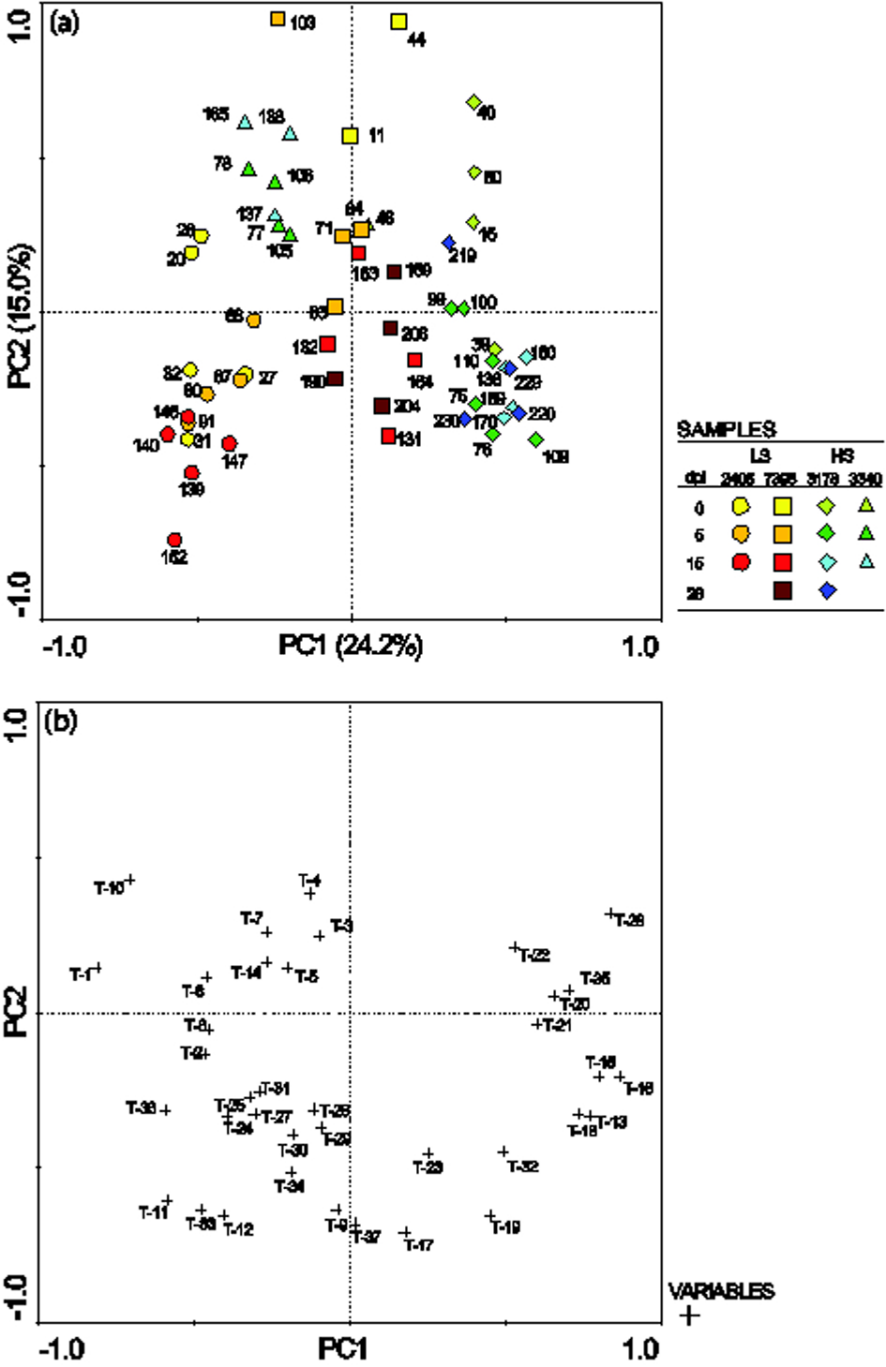
**PCA based on relative terpene composition**. (a) Sample score plot. Odd numbers: inoculated samples, even numbers: wounded samples. Less susceptible clones (LS) 2405 (circles) and 7398 (squares) are coloured in yellow-red and highly susceptible clones (HS) 3178 (diamonds) and 3340 (triangles) are coloured in green-blue. The percentages of the axes states how much of the variation the PC explain. (b) Corresponding variable loading plot. Monoterpenes (T-1-8), sesquiterpenes (T-9-19) and diterpenes (T-20-37). Further information on terpene numbering (T-#) is found in Additional file 3.

The terpene content of the constitutive samples did not indicate clear differences between high and low susceptible genotypes. Both inoculation and wounding induced terpene accumulation around the wounds but no consistent alterations of terpene composition were found. A PCA based on relative terpene composition (Figure 7) tends to separate the four genotypes from each other on the first PC but this separation did not correlate with susceptibility. At 28 dpi the accumulation of 3-carene differed between treatments (*p *< 0.01): on average *H. annosum *inoculation caused a 1200-fold increase while wounding lead to a 70-fold increase.

A limited number of contigs with significant similarity to terpene synthase (*TPS*) genes were found in the dataset (Accession number). There were three contigs with significant alignment to *(-)-α/β-pinene synthase *(*PaTPS-Pin*, [52]), each making up a separate isogroup in the dataset. One isogroup, including one isotig, showed high similarity to the previously described *(+)-3-carene synthase *gene [PaTPS-Car, 18]. Furthermore one contig had a significant BLASTx hit to *limonene synthase *(*TPS-Lim*) genes of *Picea *spp.

## Discussion

This study aimed to find associations between lower susceptibility to *Heterobasidion *spp. in Norway spruce and changes in the transcriptome and chemical profiles among host genotypes challenged with the fungus. We used unique clone material derived from fully-grown Norway spruce trees with either high or low susceptibility to *Heterobasidion *spp. as measured in a field trial [7]. We selected four genotypes at a site in central Sweden, two highly susceptible and two with lower susceptibility for these comparisons. It is well established that in the interaction between *Heterobasidion *spp. and conifers, lesion length correlates to the fungal extension but not to the host resistance measured as sapwood growth or rot extension in the wood [6,8,38]. Consequently one cannot expect a lesion extension proportional to the fungal extension under field conditions. Although, we could not detect any significant differences in lesion length between genotypes at 44 dpi, we found significantly longer lesions in the inoculated wounds compared to mock inoculations. This showed that the host trees responded differently or stronger to inoculation than to wounding.

Both terpene and phenol compositions were studied but patterns relating to inoculation or specific to genotypes with higher or lower susceptibility could only be found in the phenol fraction. There was a strong increase in terpenes after both wounding and inoculation, but no general qualitative differences. Instead, the most typical variation of terpene content was between the genotypes, without any correlation with resistance. The genotype-dependent regulation is in agreement with the work by Zeneli and co-workers [53] who report a genotype-dependent response in sapwood terpene production after treatment with methyl jasmonate. Woodward *et al. *[54] found a larger relative increase of 3-carene in less susceptible genotypes of Sitka spruce after inoculation with *H. annosum *in comparison with more susceptible genotypes. Our study showed no significant differences between genotypes of the two susceptibility levels. Nevertheless, inoculation caused a stronger 3-carene induction than wounding did, which is consistent with earlier findings in Norway spruce [20,55]. The expression levels of mono-*TPS *genes correlate with the production levels of monoterpenes in Norway spruce [52,56]. The isogroups with similarity to previously described Norway spruce mono-*TPS *genes could account for the monoterpenes identified. The absence of treatment-specific responses associated with terpenes in the chemical analyses was also reflected in the transcriptome. Although a number of sequences with significant similarity to previously described *PaTPS *genes were present in the data set, no consistent responses were found between treatments or between genotypes with high or low susceptibility. The overall picture of the regulation of terpenes based on the terpene profiles and the regulation of *TPS *genes in the transcriptome data suggests that terpenes are regulated primarily in an individual genotype-dependent manner rather than a treatment-dependent manner in this study. In Sitka spruce a small gene family of TPS-Car genes has been reported [28,57] in genotypes resistant or susceptible to white pine weevil. Also (+)-3-carene and terpinolene (another major product of TPS-Car), have recently been identified as indicators for resistance against weevils in a particular geographic region of Sitka spruce origin [58]. These studies suggests that an even more focused approach, for instance involving cloning of specific TPS-Car genomic sequences from individual genotypes, might be needed to address clone specific differences in terpene-based defence.

The constitutive phenolic composition differed between genotypes with high and low susceptibility but *H. annosum *inoculation lead to a differentiated phenolic pattern, characterized by an increase of less polar compounds, e.g. aglycones. This is in accordance with previous findings after fungal inoculations [12,14,24,26,32]. Among the constitutive phenols, astringin dimers (piceasides) are found in higher concentration in samples from genotypes with low susceptiblity. Astringin was suggested to contribute to resistance against *H. parviporum *by Lindberg *et al. *[12] since its concentration correlated negatively with hyphal growth seven days after inoculation. Stilbene dimers of astringin in Norway spruce were described the first time by Li *et al. *[59] and their ecological role has not yet been studied. However, viniferins, which are dimers of the stilbene resveratrol, showed antifungal activity in studies on grapevine and the dimers were generally more toxic than the monomer [60]. Our results indicate that the piceasides could be of importance in the defence system against *Heterobasidion *spp., but any antifungal effect remains to be shown.

Brignolas *et al. *[61] and Schmidt *et al. *[62] have suggested that activation of the biosynthetic pathways leading to flavonoids and stilbene monomers and the subsequent conversion of these into insoluble products, plays a central role in the induced defence towards wounding and fungal infection in conifers. It was suggested that resistance to *C. polonica *depends on the ability of Norway spruce to easily activate the flavonoid pathway [61]. Consequently, we were interested in whether activation of the flavonoid pathway is also of importance in the interactions with *H. annosum*. A close examination of the phenol profiles revealed that catechin accumulated significantly between 5 and 15 dpi in both *H. annosum*-inoculated and wounded bark (Figure 4). Interestingly the accumulation of free catechin was more immediate in the less susceptible genotypes in response to *H. annosum *compared with the highly susceptible genotypes. A two-way clustering of the isotigs belonging to the flavonoid biosynthetic pathway was made to reveal expression patterns. This clustering contains a number of highly similar ANR and DFR isotigs with a low level of expression (cluster 1 in Figure 5). Although no one knows exactly how many members of these gene families are to be expected in spruce it is likely that these isotigs are a reflection of the quality of the assembly. Our data set had an average read length of 305 bp (Additional file 2), which is shorter than expected due to technical problems in the cDNA synthesis and library preparation. Also we used Newbler 2.3 to assemble this data set and Kumar and Blaxter (2010) [63]reported that this version of Newbler generates assemblies with significantly lower total lengths than newer versions of Newbler. In the case with the Milkweed transcriptome the differences between Newbler 2.3 and later versions were not as drastic as reported by Kumar and Blaxter [46] suggesting that the impact of the assembler on the length of the assembly varies. Finally the samples consisted of a mixture of plant and fungal transcripts and in the absence of a spruce reference genome, this will affect the assembly of genes belonging to gene families present in both plants and fungi. Nevertheless differences between treatments can be found and validated by qPCR. The accumulation of catechin is preceded by a concomitant activation of genes in the phenylpropanoid pathway (*PAL, C4H *and *4CL*) and of genes in the epicatechin and catechin biosynthetic pathways upon wounding and *H. annosum *attack (Figure 5, 6). The strongest effect in the 454-data set is seen on the transcription of *LAR *genes, which are significantly up-regulated at 5 dpi. The up-regulation of *LAR *is accompanied by an up-regulation of the upstream *DFR*, which forms leucoanthocyanidins from which *LAR *synthesizes catechins [30,64]. Up-regulation of the competing *ANS *(*anthocyanidin synthase*), which also utilizes leucoanthocyanidines as substrates to form anthocyanidines was also observed in addition to *ANR*, an enzyme that catalyzes the synthesis of epicatechins from anthocyanidins [30,65]. In contrast, isotigs representing genes directly involved in monolignol formation (*CCR *and *CAD *do not show any significant up-regulation upon wounding or inoculation (Figure 5). This picture was confirmed by the qPCR analysis where no significant induction of *CAD *was observed (Figure 6). However the up-regulation of *PAL *and *C4H *at 5 dpi shows that the phenlypropanoid pathway is activated. Monolignol formation and lignification is a major sink for metabolites of the phenylpropanoid pathway and the modest transcriptional regulation in this pathway following wounding and *H. annosum *inoculation may suggest that a larger proportion of the metabolites are allocated to other downstream pathways such as the flavonoid pathway as indicated by the confirmed up-regulation of *LAR *and *ANR *expression. Also a recent report on Sitka spruce states that *H. annosum *inoculation or wounding do not result in any significant changes in lignin content either in bark or in sapwood, but the levels of extractable phenols do increase in the bark [66].

The level of susceptibility of Norway spruce to *Heterobasidion *infections is partly determined by the genotype [6,7]. The pattern of extractable catechin in bark differ between the clones; the two less susceptible genotypes show a significantly larger increase of free catechin between 5 and 15 days than the two highly susceptible genotypes after inoculation and wounding (Figure 4.). The qPCR results show that the transcriptional changes in the flavonoid pathway in response to *H. annosum *or wounding treatment are substantially different in 2405 and 7398, the two less susceptible genotypes (Figure 6). The qPCR data may also indicate more general differences between the clones as the expression of the PAL genes, for example, is already down-regulated at 15 dpi in wounded material of 7398 while they remain slightly up-regulated at 28 dpi irrespective of treatment in 2405 (Figure 6). The result indicates that genotype 2405 and genotype 7398 may perhaps depend on different successful defence strategies in the interaction with *Heterobasidion*.

## Conclusions

The varying dynamics in transcription chemical patterns displayed by the less susceptible genotypes suggest that there is a genotypic variation in successful spruce defence strategies against *Heterobasidion*. However, both high levels of piceasides and flavonoids in the less susceptible genotypes demonstrated the importance of the phenolic compounds in defence. Clearly an extended comparison of the transcriptional responses in the interaction with *Heterobasidion *between several independent genotypes exhibiting reduced susceptibility is needed to catalogue mechanisms of successful host defence strategies.

## Authors' contributions

MD conceived the study, did the field experiment, the analysis of phenols, the chemical analysis statistics and, assisted in the drafting of the manuscript KL conceived the study, did the field experiment, assisted in the sample preparation and the drafting of the manuscript and did the transcriptome analysis statistics, ME did the sample preparation, drafted the manuscript and assisted in the transcriptome analysis JH assisted in the terpene compound analysis, TZ did the terpene compound analysis, JA performed the qPCR, KI assisted in the sample preparation, GS assisted in the design of the study, A-KB-Kassisted in drafting the manuscript, JS conceived the study and assisted in drafting the manuscript. All authors read and approved the final manuscript

## Supplementary Material

Additional file 1 **qPCR primers used in the study**. Click here for file

Additional file 2 **454-library size and mapping metrics**. Click here for file

Additional file 3 **Denotations of phenols in Figure **3**and terpenes in Figure **7. Click here for file
