# Supplementary figures and images for: Chemical and transcriptional responses of Norway spruce genotypes with different susceptibility to Heterobasidion spp. infection
Source: BMC Plant Biol. 2011 Nov 8;11:154. doi: 10.1186/1471-2229-11-154 (PMC3240162; doi:10.1186/1471-2229-11-154)

**Additional file 1:** QPCR primers used in the study.


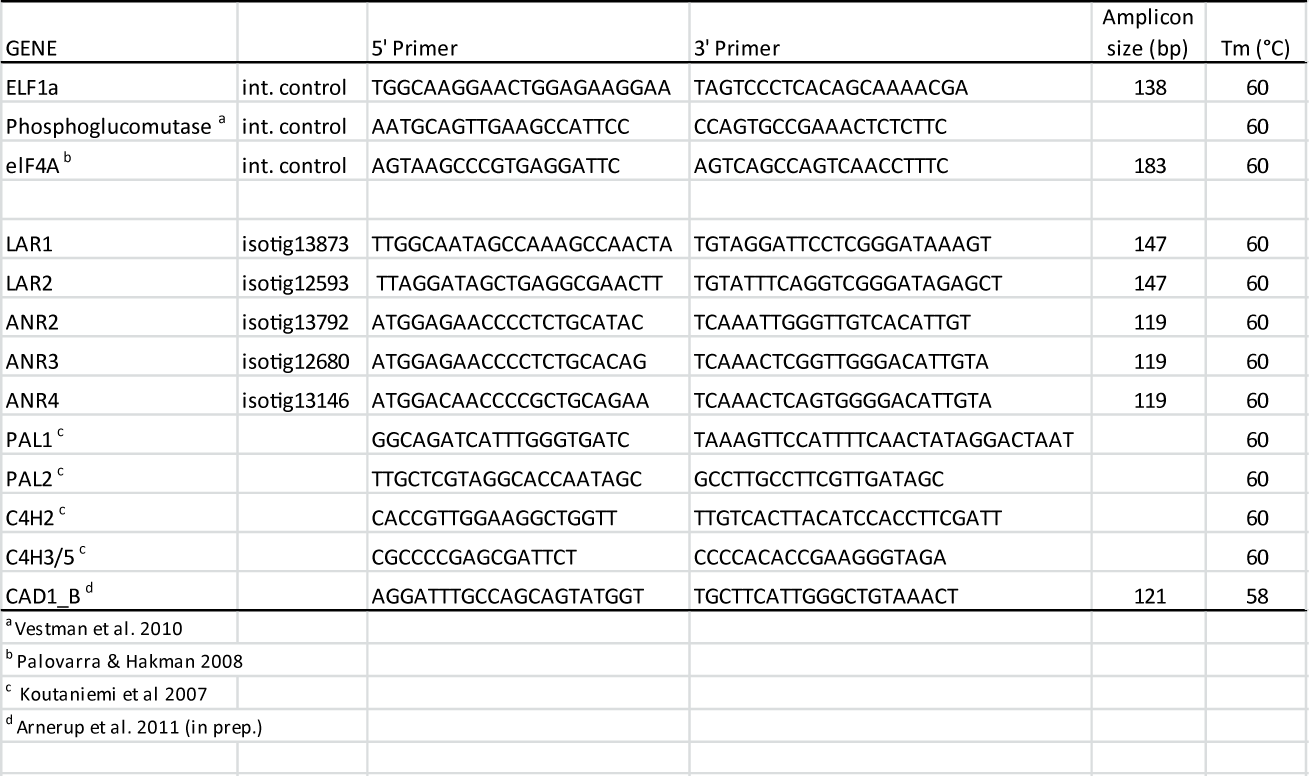

Supplement: Additional file 1 — qPCR primers used in the study. [file 1471-2229-11-154-S1.DOC]
