# Supplementary material for: Chemical and transcriptional responses of Norway spruce genotypes with different susceptibility to Heterobasidion spp. infection
Source: BMC Plant Biol. 2011 Nov 8;11:154. doi: 10.1186/1471-2229-11-154 (PMC3240162; doi:10.1186/1471-2229-11-154)
Supplement: Additional file 3 — Denotations of phenols in Figure 3and terpenes in Figure 7. [file 1471-2229-11-154-S3.DOC]

**Additional file 3:** Denotations of phenols in Fig. 3 and terpenes in Fig 7

a) Denotations of phenols in Fig. 3. uk: unknown. Only phenols for which [M-H]^-^ have been identified are include in the table, the other compounds are unknown. Compound names within parenthesis are only tentative assignments and have not been confirmed by MS/MS-spectrum or comparison with reference compounds

| P-# | Compound | [M-H]- |  | P-# | Compound | [M-H]- |
| --- | --- | --- | --- | --- | --- | --- |
| P-1 | uk | 299 |  | P-50 | (*E*)-Piceid | 389 |
| P-3 | uk | 361 |  | P-52 | (Piceaside A/ B) | 809 |
| P-6 | uk, glucoside | 325 |  | P-53 | (*Z*)-Isorhapontin | 419 |
| P-7 | uk | 417 |  | P-54 | uk | 445 |
| P-13 | Catechin | 289 |  | P-58 | (Piceaside C/D) | 823 |
| P-17 | uk | 507 |  | P-59 | (Piceaside C/D) | 823 |
| P-20 | uk | 475 |  | P-63 | Piceaside C/D | 823 |
| P-21 | uk | 495 |  | P-64 | (Kaempferol glucoside) | 477 |
| P-22 | uk | 479 |  | P-65 | (Isorhamnetin glucoside) | 447 |
| P-24 | uk, glucoside | 539 |  | P-66 | (Piceaside G/H) | 809 |
| P-25 | uk | 525 |  | P-68 | Piceaside G/H | 809 |
| P-26 | uk | 612 |  | P-69 | uk | 545 |
| P-33 | uk | 491 |  | P-73 | (Piceaside E/F) | 823 |
| P-34 | uk | 665 |  | P-74 | uk | 553 |
| P-35 | (*E*)-Astringin | 405 |  | P-75 | (Piceaside E/F) | 823 |
| P-37 | uk | 707 |  | P-76 | (Piceaside E/F) | 823 |
| P-38 | uk | 869 |  | P-78 | (Piceaside G/H) | 809 |
| P-43 | uk | 505 |  | P-81 | uk | 519 |
| P-44 | uk | 665 |  | P-82 | (*E*)-Isorhapontigenin | 257 |
| P-45 | uk | 531 |  | P-94 | uk, glucoside | 583 |
| P-47 | uk (astringin-isomer) | 405 |  | P-95 | uk | 597 |
| P-48 | uk | 855 |  | P-96 | uk | 513 |
| P-49 | (*E*)-Isorhapontin | 419 |  |  |  |  |

b) Denotations of terpenes in Fig 7. Compound names within paranthesis are tentative identifications based on comparison with spectra in the NIST-library. The terpene class is written out for unidentified compounds, MT: monoterpene, ST: sesquiterpene, STO: oxygenated sesquiterpene and DT: diterpene. The m/z of the molecular ion is included within paranthesis for the diterpenes.

| T-No | Compound ID |  | T-No | Compound ID |
| --- | --- | --- | --- | --- |
| T-1a | (-)-α-Pinene |  | T-20 | DT (272) |
| T-1b | (+)-α-Pinene |  | T-21 | DT (272) |
| T-2 | Camphene |  | T-22 | DT (272) |
| T-3 | β-Pinene |  | T-23 | DT (272) |
| T-4 | 3-Carene |  | T-24 | DT (290) |
| T-5 | Myrcene |  | T-25 | DT (290) |
| T-6a | (-)-Limonene |  | T-26 | DT (272) |
| T-6b | (+)-Limonene |  | T-27 | DT (272) |
| T-7 | β-Phellandrene |  | T-28 | DT (290, Thumbergol) |
| T-8 | MT |  | T-29 | DT (290) |
| T-9 | ST |  | T-30 | DT (286) |
| T-10 | α-Longipinene |  | T-31 | DT (272) |
| T-11 | ST |  | T-32 | DT (286) |
| T-12 | ST |  | T-33 | DT (286) |
| T-13 | ST (α-Sesquiphellandrene) |  | T-34 | DT (286) |
| T-14 | (*E*)-β-Farnesene |  | T-35 | DT (314, Methyl dehydroabietate) |
| T-15 | ST |  | T-36 | DT (286) |
| T-16 | ST (α-Cedrene) |  | T-37 | DT (288) |
| T-17 | ST |  |  |  |
| T-18 | STO |  |  |  |
| T-19 | STO (Cubenol) |  |  |  |
